# Molecular Neurobiology of Depression: PET Findings on the Elusive Correlation with Symptom Severity

**DOI:** 10.3389/fpsyt.2013.00008

**Published:** 2013-03-04

**Authors:** Donald F. Smith, Steen Jakobsen

**Affiliations:** ^1^Center for Psychiatric Research, Psychiatric Hospital of Aarhus UniversityRisskov, Denmark; ^2^PET Center, Aarhus University HospitalAarhus C, Denmark

**Keywords:** depressive disorders, symptom severity, negative emotions, positron emission tomography, brain imaging, neurobiology, neurotransmitters, reliability

## Abstract

Molecular mechanisms in the brain are assumed to cause the symptoms and severity of neuropsychiatric disorders. This review concerns the elusive nature of relationships between the severity of depressive disorders and neuromolecular processes studied by positron emission tomography (PET). Recent PET studies of human depression have focused on serotonergic, dopaminergic, muscarinic, nicotinic, and GABAergic receptors, as well as central processes dependent on monoamine oxidase, phosphodiesterase type 4, amyloid plaques, neurofibrillar tangles, and P-glycoprotein. We find that reliable causal links between neuromolecular mechanisms and relief from depressive disorders have yet to be convincingly demonstrated. This situation may contribute to the currently limited use of PET for exploring the neuropathways that are currently viewed as being responsible for beneficial effects of antidepressant treatment regimes.

Depressive disorders continue to attract much attention due to their detrimental impact on individuals and societies (Greenberg et al., 2003; McIntyre and O’Donovan, 2004; Paykel et al., 2005). Clearly, we need more effective therapeutic procedures to alleviate the disabling symptoms of depressive disorders. Much research has, therefore, been directed toward achieving that goal. Our interest here relates to the common notion that changes in the severity of depression are caused by changes in underlying neuromolecular mechanisms. In particular, much research in brain scanning by positron emission tomography (PET) has focused over the years primarily on neuroreceptors and neuronal transporter mechanisms in depressive disorders.

Positron emission tomography imaging differs from other imaging procedures by determining the dynamics of molecular processes as they occur in the living organism (Lammertsma, 2002; Laruelle et al., 2002). PET imaging uses molecules that are radiolabeled with a relatively short-lived positron-emitting nuclide. They are injected into the bloodstream so that the PET scanner can provide a digital record of the time and place of the radiolabeled molecule in the organ of interest, in this case the brain. PET brain imaging is currently used for studying neuromolecular processes in humans as well as laboratory animals (Bender et al., 2004; Smith and Jakobsen, 2007; Smith et al., 2007, 2009).

Previously, we described several challenges currently faced by PET research of depressive disorders (Smith and Jakobsen, 2009; Smith and Miller, 2010). In general, they resemble challenges described in a recent report on elusive relationships between neurobiology and symptoms in schizophrenia (Mathalon and Ford, 2012). One reason for the challenges stems from the fact that schizophrenia and depression are both heterogeneous conditions. Major symptoms of depression include hopelessness, sleep disturbance, altered appetite, lack of energy, concentration difficulties, low self-esteem, self-destructive behavior, painful bodily sensations, and suicidal ideation, with symptoms and severity differing from person-to-person. The complex nature of depression surely warns against viewing it as a single disease with a single neuromolecular cause that can be identified by a single PET scan with a single radioligand selective for a single synaptic transporter or receptor (Jones and Rabiner, 2012). Our interest in PET studies of depressive disorders has, nonetheless, induced us to prepare this account of recently published reports (Table 1), with special attention to the question of whether reliable relationships have been demonstrated between neuromolecular events and changes in depression severity. We hope that the seemingly negative findings can motivate searches in new directions using PET imaging to identify causal neuromolecular mechanisms for relief from depressive disorders.

**Table 1:** **Summary of major findings from PET brain imaging of depressive disorders for studies cited in the present review**.

Neuronal target	Radioligand	Medication status	Quantification	Diagnostic procedure	Subjects	Regions-of-interest	Outcome	Reference
Serotonin synthesis	α-[^11^C]MTrp	Not recently medicated	Patlak graphical method	SCID and HAMD	25 depressed and 24 healthy	Voxel-based, multi-ROI analysis	More in brain of depressed females than in depressed males	Frey et al. (2010)
Serotonin transporter	[^11^C]McN 5652	Not recently medicated	Arterial input function and two-tissue compartment model	SCID, HAMD, and BDI	23 depressed and 43 healthy	Anterior cingulate, amygdala, putamen, hippocampus, midbrain, and thalamus	Less binding in depressed with childhood abuse	Miller et al. (2009b)
Serotonin transporter	[^11^C]DASB	Not recently medicated	Multilinear reference tissue method	SCAN, HAMD, and BDI	20 healthy twins with or without a co-twin history of mood disorder	Orbitofrontal cortex, dorsolateral prefrontal cortex ventrolateral prefrontal cortex, anterior cingulate, caudate, putamen, thalamus, and midbrain	No reliable correlation between binding and depression scores	Frokjaer et al. (2009)
		Not recently medicated	Multilinear reference tissue method	SCID	10 unipolar depressed and 20 healthy	Thalamus	Less binding in depressed	Reimold et al. (2011)
		Not recently medicated	Multilinear reference tissue method	SCID and MADRS	16 unipolar depressed, 12 bipolar depressed, and 15 healthy	Thalamus, striatum, insula, midbrain, pregenual anterior cingulate cortex, dorsal cingulate cortex, posterior cingulate cortex, and subgenual anterior cingulate cortex	No reliable effect of depression on interaction between thalamic binding and genotype	Laje et al. (2010)
		Anti-Parkinson dopaminergic medication	Logan graphical method with reference tissue	SCID and HAMD	34 with PD and 10 health	Amygdala, hypothalamus, insula, thalamus, rostral raphe nuclei, caudal raphe nuclei, anterior cingulate cortex, posterior cingulate cortex, prefrontal cortex, caudate nucleus, putamen, and ventral striatum	No reliable difference in binding between depressed PD subjects and healthy	Politis et al. (2010)
Serotonin type 1A receptor	[^11^C]WAY-100635	Not recently medicated	Arterial input function and two-tissue compartment model	SCID and HAMD	15 remitted depressive, 13 currently depressed, and 51 healthy	Prefrontal cortices, anterior cingulate, body of the cingulate, amygdala, hippocampus, parahippocampal gyrus, insular cortex, temporal cortex, parietal cortex, and occipital cortex	More binding in depressive and depressed than in healthy	Miller et al. (2009a)
		Not recently medicated	Arterial input function and simplified reference tissue method	SCID, HAMD, and BDI	32 bipolar depressed and 47 healthy	Prefrontal cortices, anterior cingulate, body of the cingulate, amygdala, hippocampus, parahippocampal gyrus, insular cortex, temporal cortex, parietal cortex, and occipital cortex	No reliable correlation between binding and depression scores	Sullivan et al. (2009)
		Not recently medicated	Arterial input function and simplified reference tissue method	SCID, HAMD, and BDI	22 depressed and 9 healthy	Amygdala, hippocampus, parahippocampal gyrus, temporal cortex, anterior cingulate, cingulate cortex, prefrontal cortices, insular cortex, occipital cortex, and parietal cortex	More binding in depressed than healthy only for kinetic analysis by arterial input function	Parsey et al. (2010)
		Not recently medicated	Reference tissue method	SCID, HAMD, and BDI	23 depressed	Voxel-based, multi-ROI analysis	No reliable correlation between binding and therapeutic effect of psychotherapy or SSRI	Karlsson et al. (2010)
		Daily SSRI	Reference tissue method	SCID and HAMD	Nine depressed inpatients and nine healthy	Prefrontal cortex, medial frontal cortex, temporal cortex, parietal cortex, occipital cortex, anterior cingulate, insula, amygdala, hippocampus, and midbrain raphe	No reliable correlation between binding and therapeutic effect of ECT	Saijo et al. (2010a)
		Steady-state drug levels	Reference tissue method	SCID and HAMD	12 depressed and 12 healthy	Anterior cingulate cortex, orbitofrontal cortex, amygdala, hippocampus, and insula	No reliable correlation between binding and therapeutic effect of ECT	Lanzenberger et al. (2013)
		Antiepileptic drugs and some on SSRI^1^	Arterial input function and two-component model	BDI	40 with temporal lobe epilepsy	Hippocampus	High BDI score correlated with low binding in hemisphere of seizure focus	Theodore et al. (2012)
Serotonin type 1B receptor	[^11^C]P943	Not recently medicated	Multilinear reference tissue method	SCID and MADRS	10 depressed and 10 healthy	Ventral striatum and ventral pallidum	Less binding in depressed than in healthy	Murrough et al. (2011)
Serotonin type 2 receptor	[^18^F]Setoperone	>1 week antidepressant drug washout	Logan graphical method with reference tissue	SCID and HAMD	15 treatment-resistant depressed	Voxel-based, multi-ROI analysis	No reliable correlation between binding and therapeutic effect of ECT	Yatham et al. (2010)
Dopamine D_2/3_ receptor	[^11^C]FLB 457	Daily SSRI	Reference tissue method	SCID and HAMD	7 depressed inpatients and 11 healthy	Voxel-based, multi-ROI analysis	No reliable correlation between binding and therapeutic effect of ECT	Saijo et al. (2010b)
Multiple monoaminergic receptors	[^11^C]Mirtazapine	Not recently medicated	Reference tissue method	SCAN, HAMD, and BDI	17 treatment-resistant depressed and 18 healthy	Cerebral cortices, amygdala, hippocampus, putamen, caudate, and thalamus	Less binding in cortical regions of depressed than healthy	Smith et al. (2009)
MAO type A	[^11^C]Harmine	Not recently medicated	Arterial input function and two-compartment model	SCID and HAMD	18 remitted depressive, 16 depressed, and 28 healthy	Prefrontal cortex, anterior cingulate cortex, dorsal putamen, ventral striatum, thalamus, anterior temporal cortex, midbrain, and hippocampus	More binding in depressed than in healthy, but no reliable correlation between binding and therapeutic effect of SSRI	Meyer et al. (2009a)
Muscarinic type 2 receptor	[^18^F]FP-TZTP	Not recently medicated	Arterial input function and one-compartment model	SCID and MADRS	24 unipolar depressed, 16 bipolar depressed, and 25 healthy	Whole brain, anterior, posterior, and dorsal cingulated cortices, amygdala, hippocampus, ventral striatum, lateral orbital cortex, and primary visual cortex	Depending on genotype, less binding in anterior cingulated cortex of bipolar depressed than of unipolar depressed and healthy	Cannon et al. (2011)
Nicotinic acetylcholine receptors	2-[^18^F]FA-85380	Anti-parkinson drugs and 3 on antidepressant	Logan graphical method with arterial input function	SCID and BDI	22 with PD and 9 healthy	Anterior and posterior cingulate cortex, frontal lobe, parietal lobe, temporal lobe, occipital lobe, corpus callosum, hippocampus, amygdala, caudate, putamen, thalamus, midbrain, pons and cerebellum	Low binding in anterior cingulate cortex, left putamen, left midbrain, and right occipital lobe correlated with high BDI scores	Meyer et al. (2009b)
GABA type A receptor	[^11^C]Flumazenil	Initially medication-free, then SSRI in depressed	Logan graphical method with plasma input function	SCID, HAMD, BDI, and MADRS	11 depressed and 9 healthy	Anterior, ventrolateral, dorsolateral, and orbitomedial prefrontal cortex, anterior and posterior cingulate, medial and lateral temporal lobe, insular, parietal, and occipital areas, cerebellum, hippocampus, putamen, and thalamus	Lower binding in parahippocampal temporal gyri and right superior temporal gyrus in depressed than healthy. SSRI reduced binding in right lateral temporal gyrus and dorsolateral prefrontal cortex of depressed	Klumpers et al. (2010)
Phosphodiesterase type IV	[^11^C]-(*R*)-Rolipram	Not recently medicated	Logan graphical method with plasma input function	DSM-IV, HAMD, and MADRS	28 depressed and 25 healthy	Frontal, parietal, lateral temporal, occipital, medial temporal, and anterior cingulate cortices, caudate, putamen, thalamus, and cerebellum	Less binding overall in depressed than healthy, but no correlation between binding and depression scores	Fujita et al. (2012)
Amyloid and tau protein	[^18^F]DDNP	Not recently medicated^2^	Logan graphical method with reference tissue	GDS	23 MCI and 20 non-MCI middle-aged and older subjects	Hippocampus, parahippocampal areas, entorhinal cortex, posterior cingulate, lateral temporal, parietal, and frontal regions	High binding in medial temporal region correlated with high GDS score in non-MCI subjects	Lavretsky et al. (2009)
		Not recently medicated	Logan graphical method with reference tissue	SCID and HAMD	20 depressed and 18 healthy	Frontal and parietal cortices, anterior and posterior cingulate, mesial, and lateral temporal lobes	Higher binding in lateral temporal and posterior cingulate regions in depressed than in healthy	Kumar et al. (2011)
P-glycoprotein	[^11^C]Verapamil	Antidepressant drugs	Logan graphical method with plasma input function	SCID and HAMD	14 depressed and 13 healthy	Prefrontal cortex, anterior cingulate cortex, temporal lobes, amygdala, and hippocampus	Less binding in prefrontal cortex and temporal lobes in depressed than in healthy	de Klerk et al. (2009)

*^1^Personal communication from W. H. Theodore*.

*^2^Personal communication from H. Lavretsky*.

*BDI, Beck self-rating depression inventory; DSM-IV, diagnostic and statistical manual of mental disorders, 4th edition; ECT, electroconvulsive shock therapy; GDS, geriatric depression scale; HAMD, Hamilton depression rating scale; Healthy, no known psychiatric disorder; MADRS, Montgomery–Asberg depression rating scale; MCI, mild cognitive impairment; PD, Parkinson’s disease; ROI, region-of-interest; SCAN, schedules for clinical assessment in neuropsychiatry; SCID, structured clinical interview for the diagnostic and statistical manual of mental disorders*.

## PET Radioligand Binding

**Figure 1:**
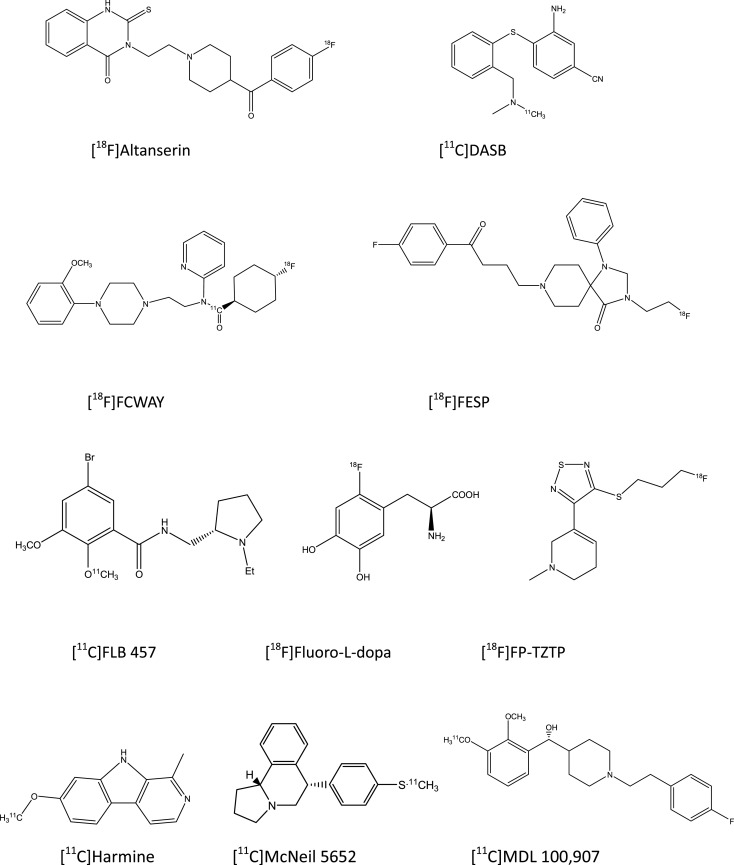

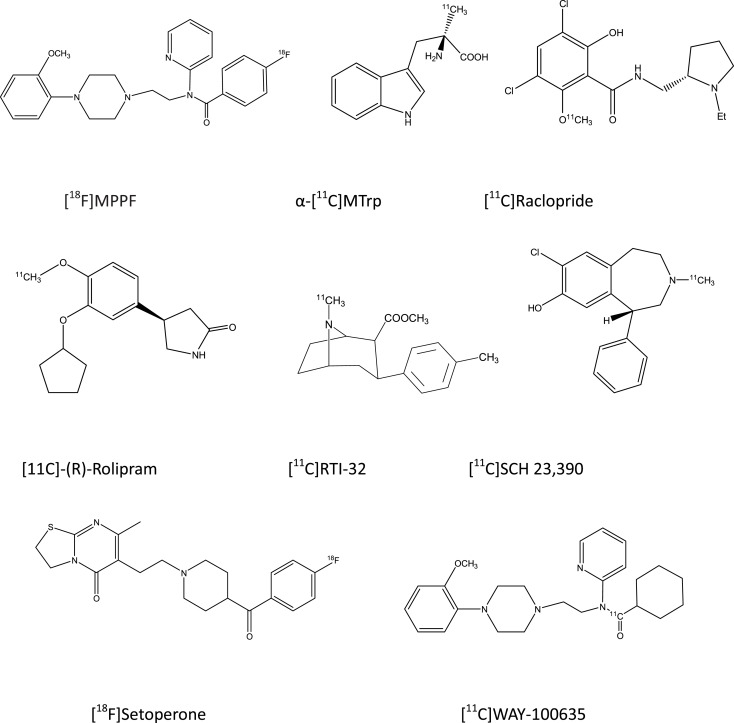
**Structural drawings of PET radioligands cited in the present review on depressive disorders**.

The basic molecular tools for PET studies are typically synthetic compounds radiolabeled with positron-emitting nuclides (Figure 1). PET radioligands travel throughout the body via the bloodstream, enter the brain, and bind to target macromolecules. The status of neuroreceptors is typically described in terms of the magnitude with which they bind the PET radioligand, expressed as binding potential or distribution volume (Innis et al., 2007). It is important to realize that PET estimates of receptor binding are a combination of three factors, namely (i) the number of neuroreceptors in a molecular conformation suited for binding, (ii) the affinity of the neuroreceptors for the radiopharmaceutical, and (iii) the concentration of endogenous neurotransmitter nearby the neuroreceptors. Thus, differences between depressed subjects and non-depressed subjects in receptor binding results in most studies from an unknown combination of pharmacodynamic factors (Laruelle, 2000; Lammertsma, 2002; Gjedde et al., 2005), which must be kept in mind in order to avoid erroneous interpretations of PET findings on neuroreceptors.

## Monoaminergic Mechanisms

### Serotonin synthesis

Disturbances in serotonin synthesis have often been assumed to contribute to depressive disorders. In a recent study, Frey et al. (2010) used α-[^11^C]MTrp to estimate the rate of serotonin synthesis in medication-free, depressed subjects, and in non-depressed, healthy controls. They hypothesized that sex differences in serotonin synthesis may underlay the well-known overrepresentation of females in lifetime likelihood of depressive disorders. Strict exclusion criteria were applied to obtain a sample of currently depressed, drug-free patients for comparison with age- and sex-matched PET data from healthy subjects retrieved from a database. Statistical analysis was carried out on normalized parametric images of the brain trapping constant for α-[^11^C]MTrp. They found that accumulation of the radioligand in selected brain regions was relatively high in medication-free, depressed females compared with medication-free, depressed males. Frey and coworkers postulated that greater disturbances of serotonin synthesis and serotonergic neurotransmission are required in males than in females for the occurrence of depression. Their notion is, however, opposed by the outcome of a study carried out previously by members of their research group (Rosa-Neto et al., 2004), in which the clinical severity of depression failed to correlate with the relative rate of serotonin synthesis in either males or females. In other studies, tryptophan depletion has been used to disrupt serotonin synthesis in males and females, but no consistent sex differences in plasma levels of the serotonin precursor, behavioral responses, or regional binding by serotonin uptake sites have been found (Moreno et al., 1999; Praschak-Rieder et al., 2005). Thus, little evidence supports the notion that sex differences in serotonin synthesis are causally related to either the likelihood or the severity of depressive disorders.

### Serotonin transport

Serotonin transport has received much attention over the years in PET studies of depressive disorders (Brust et al., 1999; Smith, 1999; Cannon et al., 2007; Meyer, 2007). Two factors have contributed strongly to such studies. Firstly, many antidepressant drugs are believe to exert their therapeutic effects by binding to the serotonin transporter and thereby preventing the removal of serotonin from the synaptic cleft (Meltzer and Lowy, 1987; Hirschfeld, 2000). Secondly, several suitable PET radioligands became available relatively early for studying regional binding of the serotonin transporter in brain (Brust et al., 1999, 2003; Houle et al., 2000). In a recent study, Miller and coworkers addressed the issue of whether disturbances in serotonin transporters provide reliable links between childhood abuse and subsequent depressive disorders (Miller et al., 2009b). They re-assessed PET data from an earlier study of adult subjects, in which [^11^C]McNeil 5652 was used to estimate the binding potential of serotonin transporters (Parsey et al., 2006a). The subjects were asked about physical and/or sexual abuse during their lifetime. The present study compared PET data from subjects that reported childhood abuse and those who reported no such experience. Binding potentials of serotonin transporters were lower in several brain regions of depressed subjects who reported childhood abuse than in those who reported no such abuse. Miller and coworkers interpreted their findings in terms of a lower density of serotonin transporters in depressed subjects who experienced childhood abuse and speculated that childhood abuse can affect the availability of serotonin transporters later in life (Miller et al., 2009b). We must note, however, that estimates of binding potentials are a composite of three factors, not just the density of receptor sites. Secondly, there is no way of knowing from their study how and when differences in the binding potential of serotonin transporters may have taken place. Therefore, their proposals appear premature and ignore several possibilities, such as differences in functional properties of serotonin transporters at birth or individual differences in effects of the experimental setting on binding potentials.

Frokjaer et al. (2009) used PET to address the issue of whether heritability affects central molecular mechanisms that may be involved in depressive disorders. They used the Danish Twin Register and the Danish Psychiatric Central Register to find subjects for the study. Their “high-risk group” consisted of twins with a co-twin having a hospital-discharge diagnosis of mood disorder, whereas their “low-risk group” had twins with a co-twin with no registered psychiatric diagnosis or personal history of mood disorder. The ultimate aim of the PET study was to identify individuals at high-risk of becoming depressed. The binding potential of serotonin transporters toward [^11^C]DASB in the dorsolateral prefrontal cortex was lower in the high-risk group than in the low-risk group, whereas no other brain regions showed reliable group differences, and the binding potentials of serotonin transporters toward [^11^C]DASB failed to correlate with scores on the Hamilton depression scale.

Reimold et al. (2011) carried out PET with [^11^C]DASB in search of links between the responsiveness of the stress hormone system and the availability of serotonin transporters in negative mood states. They studied depressed subjects who were medication-free before and during the research project. The responsiveness of the stress hormone system was assessed by measuring plasma cortisol at times after administration of dexamethasone and corticotrophic releasing hormone in a modified suppression test (Turner, 1982; Amsterdam et al., 1983). PET scans and stress tests were carried out on separate days. Binding of [^11^C]DASB was reduced in the thalamus of depressed patients compared with healthy subjects, which replicated previous findings (Reimold et al., 2011). High responsiveness in the hormone suppression test was correlated with low binding of thalamic serotonin transporters toward [^11^C]DASB. The findings serve to link negative mood states, stress responsiveness, and properties of serotonin transporters that are independent of genotype.

Politis and coworkers used [^11^C]DASB for PET to study possible relationships between binding by serotonin transporters and depressive symptoms in patients with Parkinson disease (Politis et al., 2010). Rating scales provided measures of depression severity, and cutoff points enabled classification of the Parkinson patients as being either depressed or non-depressed. Regional binding potentials of serotonin transporters in 12 brain regions were estimated by a graphical method with cerebellum gray matter as reference region. A group of healthy, non-depressed subjects was included in the study, and regional binding potentials of serotonin transporters toward [^11^C]DASB were compared between groups. Regional binding potentials were lower in all brain regions of non-depressed Parkinson patients than in healthy subjects. Similarly, the binding potentials for serotonin transporters toward [^11^C]DASB were lower in eight brain regions of depressed Parkinson patients compared with values for healthy subjects. Thus, low binding potentials of serotonin transporters were unrelated to the depression status of Parkinson patients.

Studies of serotonin transporters in depressive disorders can explore possible relationships between regional binding potentials, genotype, and symptom severity. At present, two central questions remain regarding binding potentials of serotonin transporters in depressive disorders. Firstly, is symptom severity reliably related to binding potentials? Secondly, is the genotype of serotonin transporters reliably related to binding potentials?

Murthy and coworkers performed a PET study recently to determine whether polymorphic variations in the promoter region of genes coding for the serotonin transporter, so-called S- and L-alleles (Canli and Lesch, 2007), determine regional binding potentials of serotonin transporters toward [^11^C]DASB (Murthy et al., 2010). They examined seven brain regions and estimated binding potentials by the reference tissue method. As expected (Houle et al., 2000; Ginovart et al., 2001), highest binding of [^11^C]DASB took place in raphe, putamen, amygdala, and thalamus, but binding potentials failed to be reliably related to genotype. That finding agrees with recent evidence (Reimold et al., 2011) that opposes the notion of direct causal connections between genotype and functional properties of serotonin transporters in depressive disorders (Collier et al., 1996; Mann et al., 2000; Homberg and Lesch, 2011). In addition, most studies find that symptom severity fails to correlate reliably with regional binding potentials of serotonin transporters toward [^11^C]DASB in depressed subjects (Frokjaer et al., 2009; Smith and Jakobsen, 2009; Politis et al., 2010). Thus, regional binding potentials of serotonin transporters can vary independent of genotype and of symptom severity in depressive disorders.

Laje et al. (2010) also used PET to look for links between PET findings on serotonin transporters and genotype, with focus on serotonin type 2A receptors. Their study was motivated by the outcome of a previous report on relationships between a single genotype of serotonin type 2A receptors (rs7997012) and clinical antidepressant effects of citalopram, a selective serotonin reuptake inhibitor (McMahon et al., 2006). In their recent study, Laje and coworkers determined whether binding potentials of [^11^C]DASB in several brain regions were reliably related to any of fourteen single nucleotide polymorphisms of serotonin type 2A receptors in depressed outpatients and healthy controls. The data of depressed patients and healthy volunteers were pooled in order to increase statistical power, on the assumption of no difference between diagnostic subgroups for whatever association may exist between genetic markers (genotypes) and binding potentials (phenotypes). As a result, the findings cannot indicate whether there were any reliable differences between properties of serotonin transporters in depressed versus non-depressed subjects. Special procedures, most of which are unfamiliar to the present authors, were used in the data analysis to adjust for multiple correlated comparisons. Binding potentials of [^11^C]DASB by serotonin transporters in thalamus and insula were associated with one genetic variant of serotonin type 2A receptors (rs7333412). Laje and coworkers proposed that genetic variation in serotonin type 2A receptors and either functional aspects of serotonin transporters or central serotonergic transmission affect the therapeutic response to drug-induced inhibition of serotonin transport in major depression, although their findings provided no direct empirical evidence for such claims.

### Serotonin type 1A receptor

Miller et al. (2009a) determined whether binding of [^11^C]WAY-100635 by serotonin type 1A receptors differs between healthy subjects, depressed patients, and depressive patients in remission. Their previous work had shown an increase in serotonin type 1A receptor binding in depressed patients (Parsey et al., 2006b), and the new study contained a replication attempt. None of the subjects had taken antidepressant medication for at least 6 months before PET scanning. Miller and coworkers performed PET with [^11^C]WAY-100635 to test the notion that elevated binding potentials are a molecular trait of depressive disorders. Careful diagnostic procedures and strict inclusion criteria were applied for selection of subjects. In addition, particular attention was given to the method for estimating binding potential, in light of discrepancies and controversies in previous studies (Bhagwagar et al., 2004; Parsey et al., 2006b; Moller et al., 2007; Hirvonen et al., 2008). Binding potentials of serotonin type 1A receptors toward [^11^C]WAY-100635 were, once again, higher in remitted depressed subjects and currently depressed subjects compared with healthy subjects. However, the estimates of binding potentials depended heavily on the region used as reference tissue; cerebellar white matter gave higher values for binding potentials of serotonin type 1A receptors in remitted patients than in healthy subjects, whereas cerebellar gray matter produced opposite results. Such findings suggest, surprisingly, that an important key to understanding the impact of serotonin type 1A receptors in depressive disorders resides in differences between the white and gray matter of the cerebellum.

Sullivan and coworkers studied binding of [^11^C]WAY-100635 in medication-free patients with bipolar depressive disorder compared with never-depressed, healthy subjects (Sullivan et al., 2009). A major thrust of this study concerned comparisons between methods for estimating the binding potential of serotonin type 1A receptors toward the PET radioligand. They included arterial blood sampling with analysis of metabolites and free plasma fraction for estimates of binding potentials of regional serotonin type 1A receptors. In addition, estimates of binding potentials were obtained by a reference region method, and the serotonin type 1A gene was genotyped to see whether genetic factors affect 5-HT1A binding in bipolar disorder. Values for regional binding potentials of serotonin type 1A receptors toward [^11^C]WAY-100635 were log transformed, and statistical analysis was then carried out by a linear mixed-effects model. Estimates of regional binding potentials, based on plasma data, were higher in bipolar depressed patients than in never-depressed subjects, due mainly to relatively high values in depressed, bipolar males, along with lower free plasma fractions in depressed, bipolar subjects compared with never-depressed subjects. It is noteworthy that values for regional binding potentials of serotonin type 1A receptors toward [^11^C]WAY-100635 failed to correlate reliably with the clinical condition of subjects, and no reliable relationships were noted between diagnostic groups for [^11^C]WAY-100635 binding and C(-1019)G promoter polymorphisms. Similarly, estimates of binding potentials by a reference region method provided no reliable difference between bipolar depressed patients and never-depressed subjects.

Parsey et al. (2010) carried out another PET study with [^11^C]WAY-100635 to examine possible sources of discrepancies in previous studies concerning whether regional binding potentials of serotonin type 1A receptors are higher or lower in major depressive disorders than in non-depressed subjects. As noted above (Sullivan et al., 2009), regional binding potentials of serotonin type 1A receptors toward [^11^C]WAY-100635 were reduced in depressed subjects compared with non-depressed subjects when reference tissue was cerebellar gray matter, whereas opposite findings were obtained with the use of cerebellar white matter as reference tissue. The marked sensitivity of estimates of radioligand binding by serotonin type 1A receptors on selection of cerebellar tissue complicates the use and interpretation of reference region methods for PET studies of depression with [^11^C]WAY-100635.

Karlsson and coworkers determined whether the binding potential of serotonin type 1A receptors is affected by psychotherapy and/or antidepressant drug treatment (Karlsson et al., 2010). Using a randomized design, they treated depressed subjects from a previous PET study (Hirvonen et al., 2008) with either fluoxetine or short-term psychodynamic therapy. Brain imaging with [^11^C]WAY-100635 was done before and after treatment, along with symptom assessment by the Hamilton Depression Rating Scale. They used the reference region method based on cerebellar white matter and found increased binding potentials of serotonin type 1A receptors toward [^11^C]WAY-100635 in cortical regions in the group given psychotherapy compared with those given fluoxetine, despite the fact that improvement of depression was similar in the two groups. Compared with healthy subjects, post-treatment binding potentials of serotonin type 1A receptors were elevated in the psychotherapy group but not in the fluoxetine group. Karlsson and coworkers view their findings as direct evidence for a specific neurotransmitter mechanism involved in the neurobiology of psychotherapy, perhaps unrelated to therapeutic response.

Saijo et al. (2010a) did a study to see whether antidepressant treatment affects the binding potential of serotonin type 1A receptors. In their case, the antidepressant treatment consisted of electroconvulsive therapy (ECT). Using PET with [^11^C]WAY-100635, brain imaging took place before and after ECT in a group of depressed inpatients. Kinetic data analysis was carried out using the reference region method based on cerebellar gray matter. Never-depressed subjects underwent PET scanning with [^11^C]WAY-100635 on one occasion to provide measures of regional binding by serotonin type 1A receptors for comparison with values in depressed inpatients. The patients continued to receive selective serotonin reuptake inhibitors during ECT. Depression improved markedly in all patients given ECT between the first and second PET scans, but no reliable changes occurred in regional binding potentials of serotonin type 1A receptors. Compared with never-depressed subjects, reduced binding potentials of serotonin type 1A receptors were noted in the midbrain raphe region both before and after completion of antidepressant therapy. The findings agree with a previous report on the lack of direct relationships between binding by serotonin type 1A receptors and negative mood state (Bhagwagar et al., 2004).

Lanzenberger et al. (2013) also determined by PET whether ECT alters [^11^C]WAY-100635 binding in brain regions. They scanned treatment-resistant depressed patients twice before ECT begun, so as to establish the test-retest validity of the procedure and, thereby, to improve the initial characterization of serotonin type 1A receptors. The patients remained in steady-state drug treatment during the study. Cerebellar gray matter, excluding the vermis, served as reference tissue for estimating the binding potential of serotonin type 1A receptors. ECT lowered the binding potential of serotonin type 1A receptors toward [^11^C]WAY-100635 throughout the brain, without region-specific effects. Of particular importance for the present review is the fact that no reliable correlations were noted between the magnitude of treatment-induced changes in radioligand binding by serotonin type 1A receptors and changes in scores on the Hamilton depression scale.

Theodore et al. (2007, 2012) followed-up a previous PET study on possible dysfunctions of serotonin type 1A receptors in depressive disorders in patients with temporal epilepsy. In their new study, they looked for relationships between the binding potential of hippocampal serotonin type 1A receptors toward [^11^C]WAY-100635 and depression in epileptic patients. They used metabolite-corrected plasma input functions in their estimates of binding potentials, and found that high scores on the Beck depression self-rating inventory were correlated with low hippocampal binding of [^11^C]WAY-100635 in the hemisphere on the side of the seizure focus. We can only wonder about the potential clinical importance of these findings, while we wait for them to be replicated to judge their validity.

A major controversy in PET studies of serotonin type 1A receptors currently centers around differences noted between studies of regional binding of [^11^C]WAY-100635 in depression (Shrestha et al., 2012). Uncertainties may stem from a range of factors, including choice of reference region, measurement of radiometabolites, altered affinity state of receptors, and relatively small samples (Shrestha et al., 2012). There is, nonetheless, general agreement that published findings fail to demonstrate reliable relationships between the severity of depression and [^11^C]WAY-100635 binding. That situation in itself casts doubt on the notion that PET studies of serotonin type 1A receptors can disclose the neurobiological basis of depressive disorders. In view of radiation doses received by subjects in PET studies along with the relatively great expense of such studies, perhaps the time has come to abandon the apparently futile search for confirmatory evidence that regional binding potentials of serotonin type 1A receptors toward [^11^C]WAY-100635 are critical for the course of psychic depression.

### Serotonin type 1B receptor

Murrough and coworkers carried out the first PET study on serotonin type 1B receptors in depressive disorders (Murrough et al., 2011). They estimated the binding potential of [^11^C]P943 (Nabulsi et al., 2010) in brain regions rich in serotonin type 1B receptors, namely ventral striatum and ventral globus pallidus (Sari, 2004). The study used healthy subjects and medication-free subjects in prolonged episodes of major depressive disorder. Kinetic data analysis consisted of a multiple reference tissue method (Ichise et al., 2003) with cerebellum as reference region. PET data were expressed as the ratio between binding potentials of serotonin type 1B receptors in ventral striatum versus ventral globus pallidus. That ratio was markedly lower in both hemispheres of subjects with major depressive disorder than in healthy subjects, but it failed to correlate with clinical or demographic items. In addition, we wish to point out that presenting PET data only as ratios precludes gaining an understanding of the neuromolecular source of the alleged differences between healthy and depressed subjects in receptor binding in brain regions.

### Serotonin type 2 receptor

Yatham and coworkers examined the status of serotonin type 2 receptors before and after a series of right unilateral ECT in depressed subjects who were refractory to previous antidepressant therapies (Yatham et al., 2010). Needless to say, ECT provides one of the most powerful antidepressant procedures by which to explore possible relationships between depression severity and neuroreceptor binding (Minichino et al., 2012). The number of ECTs administered to each depressed subject was based on clinical response, with a maximum of eight treatments. Psychotropic drugs other than benzodiazepines for sleep were discontinued at least 1 week prior to PET scanning with [^18^F]setoperone. Estimates of the binding potential of serotonin type 2 receptors in the cerebral cortex toward [^18^F]setoperone were obtained by a graphical reference tissue method. Depressed subjects who were included in this study had relatively high scores on the Hamilton depression rating scale, indicative of their severe condition. Binding potentials of serotonin type 2 receptors in several cortical regions, including medial prefrontal cortex and parahippocampal gyri, were lower after ECT than before treatment, but the magnitude of clinical improvement failed to correlate reliably with changes in binding potentials.

The lack of a reliable relationship between the clinical condition of depressed patients given ECT and regional binding of [^18^F]setoperone by serotonin type 2 receptors challenges the notion of causal links between depressive disorders and that receptor system.

### Dopamine D_2/3_ receptor

Saijo et al. (2010b) used [^11^C]FLB 457 for PET to determine whether ECT affects the binding of extrastriatal dopamine D_2/3_ receptors in depressed subjects. They carried out PET scanning before and after ECT in depressed patients who were allowed to continue taking antidepressant medication during the study. A group of never-depressed, healthy subjects were included in the study; they were scanned once in order to obtain baseline estimates of binding of [^11^C]FLB 457 by dopamine D_2/3_ receptors for comparison with depressed inpatients. ECT reduced the severity of depression in all patients, but binding potentials of dopamine D_2/3_ receptors toward [^11^C]FLB 457 in patients failed to be reliably related to improvement in their clinical condition. Binding potentials of dopamine D_2/3_ receptors toward [^11^C]FLB 457 were lower in right anterior cingulate after ECT than before treatment, but no reliable differences were noted between depressed patients and never-depressed subjects.

Normalized parametric images were used by Saijo and coworkers in their analysis of dopamine D_2/3_ receptor binding. That procedure fails, however, to provide quantitative estimates for statistical comparisons. Nevertheless, it is noteworthy once again that the clinical condition of depressed subjects was unrelated to [^11^C]FLB 457 binding by dopamine D_2/3_ receptors.

### Multiple monoamine receptors

Smith and coworkers radiolabeled mirtazapine, a multitarget antidepressant compound (Millan, 2006, 2009), for PET in treatment-resistant, medication-free, depressed subjects, and in never-depressed, healthy subjects (Smith et al., 2009). The rationale for the study centered on the notion that treatment-resistant depression may involve dysfunction of several neuroreceptor systems that may be assessed simultaneously by a multitarget PET radioligand. Binding potentials of receptors toward [^11^C]mirtazapine were lower in cortical regions of treatment-resistant, depressed subjects than in healthy subjects, and depression scores were correlated inversely with binding potentials in frontal, temporal, and occipital cortical regions. However, the multiple pharmacological actions of [^11^C]mirtazapine on central receptor systems preclude statements on the relative role of specific neuromolecular mechanisms in differences detected by PET between depressed and healthy subjects. Another drawback of the study concerns differences in the specific activity of the radioligand administered to healthy versus depressed subjects. That factor was dealt with by *post hoc* regression analysis, which is a procedure with many pitfalls (Vul et al., 2009; Simmons et al., 2011).

### Monoamine oxidase type A

Meyer and coworkers explored possible relationships between depressive disorders and regional binding of [^11^C]harmine, a PET radioligand for studying monoamine oxidase type A (Meyer et al., 2009a). They determined whether therapeutic effects of antidepressant drug treatment affects binding of [^11^C]harmine by monoamine oxidase type A, and whether recurrence of depression is associated with the level of [^11^C]harmine binding in brain regions. To determined whether successful treatment with an antidepressant drug affects central binding of [^11^C]harmine, they PET scanned depressed subjects twice, once before treatment, and again after 6 weeks of treatment with a selective serotonin reuptake inhibitor. It is noteworthy for the present review that beneficial clinical effects of antidepressant treatment failed to reliably affect regional binding of [^11^C]harmine in the brain. Some relationships were noted, nevertheless, between binding of [^11^C]harmine and depression. For example, [^11^C]harmine binding was elevated in brain regions of depressed subjects and of subjects who had recovered from depression compared with never-depressed subjects, and recurrence of depression was associated with elevated [^11^C]harmine binding.

This study was carried out with meticulous attention to detail in screening subjects, scanning procedures, and kinetic data analysis. In our view, the failure of beneficial antidepressant treatment to affect [^11^C]harmine binding as measured by PET dissociates depressive psychopathology from central levels of monoamine oxidase type A. The relationship reported between elevated activity of monoamine oxidase type A in some brain regions and recurrence of depression was statistically significant without correction for multiple comparisons, but inspection of the data indicates marked overlap between groups, which tends to call that finding into question.

## Non-Monoaminergic Mechanisms

### Muscarinic type 2 receptor

Cholinergic neurotransmission has a widespread distribution in the brain and the parasympathetic nervous system (Mineur and Picciotto, 2010; Wevers, 2011). Cannon and coworkers combined PET brain imaging and genotyping in their study of central cholinergic processes in depressive disorders (Cannon et al., 2011). They assessed possible relationships between six single nucleotide polymorphisms of the cholinergic muscarinic type 2 receptor gene and binding of the high-affinity cholinergic agonist, [^18^F]FP-TZT. Their study had several strong points. For example, they included an independent test of a previous finding (Cannon et al., 2006), applied strict exclusion criteria with regard to the use of nicotine and psychotropic drugs by their subjects, and used appropriate statistical procedures with corrections for multiple comparisons (Curran-Everett, 2000; Nieuwenhuis et al., 2011). They found that differences in the degree of binding of [^18^F]FP-TZTP in the anterior cingulate cortex of bipolar depressive versus healthy subjects depended on the allelic composition of the single nucleotide polymorphism (rs324650), whereas no such differences were noted for [^18^F]FP-TZTP binding between healthy subjects and subjects with unipolar depressive disorder. Cannon and coworkers interpreted their finding of relatively low [^18^F]FP-TZTP binding in bipolar disorder in relation to the nucleotide sequence coding for the muscarinic type 2 receptor. In our view, that is a truly bold hypothesis on the basis of a single study.

### Nicotinic receptor

Nicotinic receptors contribute to central molecular mechanisms ranging from nicotine dependence to severe cognitive dysfunction (Dani and Bertrand, 2007; Romanelli et al., 2007). Meyer et al. (2009b) reasoned that disturbances in nicotinic receptors may contribute to symptoms of depression in patients with Parkinson disease. To test that notion, they carried out PET with [^18^F]FA-85380 in Parkinson patients and healthy subjects. All subjects were non-smokers, which ruled out nicotine dependence as a confounding factor (Brody et al., 2006). Depression in Parkinson patients was determined by a self-report questionnaire, and PET data were analyzed by region-of-interest and voxel-based procedures. As expected (Perry et al., 1995), binding by nicotinic receptors was reduced in several brain regions of Parkinson patients compared with healthy subjects. In addition, depression scores in Parkinson patients were correlated inversely with binding of [^18^F]FA-85380 in several brain regions. Meyer and coworkers speculated that their PET procedures may enable early detection of nicotinic alterations in brain regions of Parkinson patients at risk of developing depressive disorders.

### Gamma amino butyric acid (GABA) type a receptor

GABAergic mechanisms are currently receiving much attention in research on psychic disorders (Rudolph and Knoflach, 2011). Klumpers and coworkers used [^11^C]flumazenil for PET to see whether binding of central GABA_A_ receptors differs between depressed and healthy subjects (Klumpers et al., 2010). In addition, they determined whether antidepressant drug treatment affects GABA_A_ receptor binding. The findings depended heavily on the method used for PET data analysis. Voxel-based data analysis indicated that antidepressant drug treatment reduced [^11^C]flumazenil binding in right lateral temporal gyrus and dorsolateral prefrontal cortex of depressed patients. In contrast, region-of-interest analysis revealed no reliable differences of antidepressant treatment on [^11^C]flumazenil binding, no differences in [^11^C]flumazenil binding between depressed and healthy subjects, and no reliable correlations between regional receptor binding potentials and depression scores. In a selected sample of patients, a relationship was found between reduced binding of [^11^C]flumazenil at certain brain sites and lack of neuroendocrine suppression in a dexamethasone-suppression test. Klumpers and coworkers interpreted their findings in terms of putative deficiencies in inhibitory GABAergic mechanisms.

### Phosphodiesterase type 4

Fujita and coworkers used [^11^C]-(*R*)-rolipram for PET to probe the cAMP second messenger system in healthy subjects and depressed patients (Fujita et al., 2012). Rolipram was originally developed as an antidepressant drug to inhibit phosphodiesterase type 4 and thereby reduce metabolism of cAMP (Wachtel, 1982, 1983; Zanotti-Fregonara et al., 2011). As usual (Gjedde et al., 2005), estimates of radioligand binding in brain regions depended on the density and the activity of the enzyme, as well as the concentration of other compounds that compete at the receptor site. Fujita and coworkers found that [^11^C]-(*R*)-rolipram binding was reliably lower throughout the brain of depressed patients than in healthy subjects, with no reliable correlations between radioligand binding and the severity of depressive symptoms.

### Amyloid and tau proteins

Pathological changes in the brain consisting of amyloid plaques and neurofibrillar tangles typically herald cognitive disturbances in Alzheimer disease (Vardy et al., 2006; Golde et al., 2010; Small et al., 2012). Lavretsky et al. (2009) used [^18^F]DDNP to see whether amyloid plaques and neurofibrillar tangles are also present in the brain of middle-aged and older subjects with mild-to-moderate depressive disorders, with and without mild cognitive impairment. None of the subjects had required antidepressant drug treatment. Depressive symptoms in subjects with mild cognitive impairment were associated with increased binding of [^18^F]DDNP in lateral temporal regions, whereas symptoms of depression in subjects without mild cognitive impairment were associated with increased binding of [^18^F]DDNP in medial temporal regions. Thus, amyloid plaques and neurofibrillar tangles were identified by PET in elderly, mildly depressed subjects, regardless of their cognitive condition.

Kumar and coworkers also used [^18^F]DDNP for PET to explore possible links between depressive disorders and amyloid plaques and neurofibrillar tangles (Kumar et al., 2011). They recruited non-demented subjects with late-life depression, and compared them to non-depressed, age-matched subjects. Binding of [^18^F]DDNP in several cortical brain regions was higher in the group with late-life depression than in the age-matched, non-depressed group.

### P-glycoprotein

The blood-brain barrier limits the bi-directional passage of molecules, while the removal of certain molecules from the brain is enhanced by the P-glycoprotein efflux pump. Verapamil is a calcium channel blocker that is removed from the brain by the P-glycoprotein pump, and the drug can be radiolabeled with a positron-emitting nuclide and used to study the P-glycoprotein pump (Bartels et al., 2010). de Klerk et al. (2009) examined possible relationships between the P-glycoprotein pump and depressive disorders. They used [^11^C]verapamil for PET brain imaging in a group of severely depressed subjects receiving various antidepressant drugs and in a group of non-depressed, unmedicated subjects. The binding of [^11^C]verapamil in the whole brain failed to differ reliably between groups, while regional data analysis indicated that levels of [^11^C]verapamil were lower in prefrontal cortex and temporal lobes of depressed subjects than in healthy controls. de Klerk and coworkers interpreted their finding in relation to the possible impact of long-term usage of antidepressant drugs and/or treatment-resistant depression on the P-glycoprotein pump.

The diversity of recent PET studies on non-monoaminergic mechanisms in depressive disorders precludes general statements concerning the findings. [^18^F]DDNP was used in two recent studies that found an association between binding by amyloid plaques and neurofibrillar tangles in temporal lobes of elderly subjects with mild-to-moderate depression. That finding concurs with reports on neuropathology in temporal cortex of depressed subjects (Saylam et al., 2006; Vasic et al., 2008) and supports the well-known relationship between depressive disorders and Alzheimer’s disease (Caraci et al., 2010; Frisardi et al., 2011). Cholinergic mechanisms have also been implicated in both depressive disorders and Alzheimer’s disease (Mineur and Picciotto, 2010; Fisher, 2012), which may relate to the differences observed in binding of [^18^F]FP-TZTP and [^18^F]FA-85380 between healthy subjects and subjects with depressive disorders. With regard to GABAergic neurotransmission, much evidence implicates disturbances of function in depression as well as other neuropsychiatric disorders (Rudolph and Knoflach, 2011). The recent PET findings with [^11^C]flumazenil are of interest in that respect (Klumpers et al., 2010), despite the fact that beneficial effects of antidepressant treatment were associated with a further reduction of radioligand binding. Obviously, we have much to learn regarding whether non-monoaminergic mechanisms can be reliably linked with depressive disorders by PET brain imaging.

## General Discussion

The search continues for PET radioligands that can disclose causal links between the binding properties of central neuromolecular processes and the clinical condition of people suffering from depressive disorders. Our account of PET findings presented in this review is both critical and harsh, based on our serious concern regarding the current lack of clear-cut relationships between neuromolecular processes as measured by PET and changes in the severity of depression. In particular, beneficial effects of potent antidepressant treatments have typically failed to affect neuroreceptor binding of PET radioligands in subjects who were depressed at the start of the study, but who experienced clear-cut reductions in symptom severity. We are aware that lack of effect of beneficial antidepressant treatment on receptor binding in depressed subjects can be said to reflect a so-called “trait variable,” but we fail to see the value of that notion since it has no impact on treatment strategy. What is more, the notion of a “trait variable” as a stable, neuromolecular abnormality fails to take into account the possibility that the foreign experience of being constrained in a PET scanner and having a radioactive substance injected into a vein may affect brain function differently in depressed subjects than in healthy volunteers. If that were to occur, then the PET findings would reflect a so-called “state variable” rather than a “trait variable.”

The failure of changes in the clinical condition of depressed subjects to be reliably linked with changes in binding of PET radioligands in the living brain adds to the disappointment that currently surrounds the field (Jones and Rabiner, 2012). Sadly, studies carried out by PET with available radioligands have neither proved nor refuted conclusively any hypothesis concerning causal connections between molecular neurobiology and the severity of depression. The failure of PET to demonstrate reliable effects on neuroreceptors in depressed subjects receiving potent antidepressant treatments may indicate that neuronal systems probed by currently available positron-emitting radioligands are remarkably stable. If so, then the success of PET scanning in detecting causal relationships between symptoms of depression and molecular neurobiology may require the invention of further positron-emitting radioligands along with new scanning procedures that can detect dynamic variations in neuromolecular processes that remain unknown. The richness of human emotions, thoughts, and actions, plus the complexity of molecular events in the human brain, caution against expecting PET brain imaging to provide rapid progress toward improving the treatment of depressive disorders.

## Conflict of Interest Statement

The authors declare that the research was conducted in the absence of any commercial or financial relationships that could be construed as a potential conflict of interest.
